# Network-guided interaction mining for the blood pressure phenotype of unrelated individuals in genetic analysis workshop 19

**DOI:** 10.1186/s12919-016-0052-7

**Published:** 2016-10-18

**Authors:** Adeline Lo, Michael Agne, Jonathan Auerbach, Rachel Fan, Shaw-Hwa Lo, Pei Wang, Tian Zheng

**Affiliations:** 1 Department of Politics, Princeton University, 130 Corwin Hall, Princeton, NJ 08544-1012 USA; 2 Department of Statistics, Columbia University, New York, 10027 USA; 3 Department of Genetics and Genomic Sciences, Icahn School of Medicine at Mount Sinai, New York, 10029 USA

## Abstract

Interactions between genes are an important part of the genetic architecture of complex diseases. In this paper, we use literature-guided individual genes known to be associated with type 2 diabetes (referred to as “seed genes”) to create a larger list of genes that share implied or direct networks with these seed genes. This larger list of genes are known to interact with each other, but whether they interact in ways to influence hypertension in individuals presents an interesting question. Using Genetic Analysis Workshop data on individuals with diabetes, for which only case-control labels of hypertension are known, we offer a foray into identification of diabetes-related gene interactions that are associated with hypertension. We use the approach of Lo et al. (Proc Natl Acad Sci U S A 105: 12387-12392, 2008), which creates a score to identify pairwise significant gene associations. We find that the genes *GCK* and *PAX4*, formerly known to be found within similar coexpression and pathway networks but without specific direct interactions, do, in fact, show significant joint interaction effects for hypertension.

## Background

Hypertension is a well-studied genetic disease, particularly in the identification of genes marginally associated with the disease. When using high-throughput data such as genome-wide association studies or sequencing data we must also consider interactions between genes, which can simultaneously and dramatically increase the number of dimensions required for evaluation, as well as the chance of false positives. Reduction of dimensionality can be preliminarily conducted through literature-based confirmations of biological relations and possible interactions of genes, and focusing on these sets of genes first. Laboratory and data analysis have developed biological and functional interactions between some of these identified genes. This paper seeks to further the network knowledge of genes that interact to affect hypertension.

Using a “seed” set of 15 genes found to be theoretically associated with type 2 diabetes in the literature, we expand on this seed set with genes known to broadly interact with these seed genes (although specific information on their interactions to influence type 2 diabetes is unknown) to create our full gene list. We then explore pairwise associations in our full gene list by providing a systematic exploration of all significant pairwise associations (potentially expanding on edges in the literature’s drawn network of these genes). Because the Genetic Analysis Workshop (GAW) data is on individuals with type 2 diabetes but only the phenotype for blood pressure is known, we use network information on genes interacting for type 2 diabetes to identify novel gene interactions for hypertension. We believe that, for these individuals, underlying diabetes mechanisms drives variations in hypertension status. We use this study to identify potential association of blood pressure with diabetes genes in this data set.

## Methods

### Seed genes from literature

To build the original set of genes theoretically associated with hypertension, we turned to the Online Mendelian Inheritance in Man (OMIM) [1]. Using type 2 diabetes mellitus as the search term (#125853), we found a list of 15 genes known to be related to diabetes type 2 (Table 1). These were then used in GeneMANIA to retrieve genes connected to them.

**Table 1:** Seed gene list

Seed Gene List	
*IGF2BP2*	*IRS2*
*PPARG*	*SLC2A4*
*GCK*	*HNF1B*
*KCNJ11*	*GCGR*
*ABCC8*	*RETN*
*MAPK8IP1*	*PDX1*
*MTNR1B*	*PPARG*
*IPF1*	

All seed genes taken from OMIM

### Interaction network from literature

We supplied all 15 genes to the online portal of GeneMANIA [2], an online database of connections, including known biological pathways, between genes reported in the literature so far. The seed genes were used to retrieve genes that are connected to them. Each connected gene was scored based on the nature and strength of evidence of all the connection instances it had with all seed genes. We chose the 20 top-scored genes to expand the previous set of 15 seed genes, resulting in a final list of 22 seed and expanded genes. We denoted this list of genes as $V=\left({v}_1,\dots,\;{v}_k\right),\;k\in \left\{1,\dots, 22\right\}$. We also retrieved the interaction network, denoted as $E={\left[{e}_{ij}\right]}_{k\times k}$.

### Hypertension phenotype

The data set under consideration was the GAW19 [3] unrelated individuals data with type 2 diabetes. The phenotype available for analysis, however, was hypertension. Subjects were coded as case or control phenotypes in parallel to the rules used in the GAW19 family data set, whereby cases were defined as individuals with systolic blood pressure (SBP) >140 mm Hg, diastolic blood pressure (DBP) > 90 mm Hg, or who were on antihypertensive medication. Satisfying any one of these three criteria was sufficient to make them a case. Controls measured as SBP ≤140 mm Hg, DBP ≤90 mm Hg, and were not on antihypertensive medication.

### Pairwise network scoring

We use the approach in Lo et al. [4] to score the marginal and joint effects. Because we have 22 genes, we have $\frac{\left(22*21\right)}{2}=231$ gene pairs.

To measure the joint effects of two single nucleotide polymorphisms (SNPs), we use the measure *v*:

$$ v={\displaystyle \sum_{s=1}^{3^k}}{\left(\frac{n_{D,s}}{n_D}-\frac{n_{U,s}}{n_U}\right)}^2 $$

where ${n}_{D,s}$ and ${n}_{U,s}$ are counts of cases and controls in each genotype (element) *s*, ${n}_D$ and ${n}_U$ are the total number of cases and controls under the study, and $k\in \left\{1,\dots, 22\right\}$.

To measure the amount of interaction between two genes, ${g}_i$ and ${g}_j$: for every pair of SNPs $\left({i}_d,\;{i}_e\right)$ from each gene $i$ and gene $j$ define the SNP-wise interactions as the ratio of incremental interactions versus the maximum of the two marginal effects:

$$ r\left({i}_d,\ {j}_e\right)=\frac{v_{i_d,{j}_e}-{v}_{i_d}\mathsf{V}\ {v}_{j_e}}{v_{i_d}\mathsf{V}\ {v}_{j_e}} $$

where $\vee$ represents the maximum of two values, and $r\left({i}_d,\;{j}_e\right)$ is the relative amount of interactions of two SNPs with respect to their marginal effects. The amount of interactions between two genes $i$ and $j$ is defined as the average of all SNP-wise ratios possibly formed from these two genes and is denoted as:

$$ {R}_{ij}=\frac{{\displaystyle {\sum}_{d=1}^{m_i}}{\displaystyle {\sum}_{e=1}^{m_j}}r\left({i}_d,\ {j}_e\right)}{m_i{m}_j} $$

and called the “mean interaction ratio,” or “mean-ratio” or “R-statistic.”

For each gene pair, we also define the “average maximum marginal $v$” or “M-statistic” as:

$$ {M}_{ij}=\frac{{\displaystyle {\sum}_{d=1}^{m_i}}{\displaystyle {\sum}_{e=1}^{m_j}}\left({v}_{i_d}\vee {v}_{j_e}\right)}{m_i{m}_j} $$

From the above steps, we obtain a set of 231 total pairwise interactions, $\left\{\left({M}_{ij},\;{R}_{ij}\right);1\le i<j\le 22\right\}$, corresponding to all possible gene pairs.

To establish significance, we applied 1000 permutations of the case-control outcomes in order to determine the null distribution of the ratio and maximum. Permutations are used to determine significance between gene interactions.

## Results

### Retrieved pairwise network scores

Results of all pairwise SNP interactions resulted in 41 pairwise SNPs with statistically significant joint effects (significant $v$ scores). However, this is based on theoretical results (Table 2 lists the SNPs and their respective joint effect scores). Indeed, these are only amongst SNP interactions; to determine whether genes are significantly associated with other genes, we average across SNP–SNP interactions between one given gene and another given gene. Given the rare variant-heavy nature of the GAW19 data set, marginal and joint association scores were very low; this is not surprising given the rarity of the variants. However, even with the rare variant problem, one set of joint gene interactions was found after 1000 permutations, between gene *GCK* and gene *PAX4* at the 95 % significance level.

**Table 2:** Top returned ratio of pairwise and marginal effects

SNP1	SNP2	Joint score (normalized)
7	647	2.920
85	409	4.165
85	426	3.645
85	441	2.870
85	614	3.003
96	603	2.773
125	647	2.798
138	647	2.705
150	647	3.104
215	647	2.781
221	833	3.023
344	620	2.898
347	620	2.829
357	620	2.885
358	647	2.817
344	751	2.880
347	751	2.864
357	751	3.021
375	714	3.102
387	451	3.169
387	452	2.771
414	647	2.765
441	620	2.860
441	647	2.737
451	614	2.906
451	620	2.857
441	714	2.719
441	716	2.732
451	714	3.114
451	733	2.742
452	714	2.899
451	747	3.561
451	754	3.987
451	759	4.011
452	747	3.488
452	754	3.614
452	759	3.640
441	812	3.307
451	872	2.786
647	698	3.673
647	829	3.405

## Discussion

The main results are 41 pairwise SNPs that demonstrate statistically significant joint effects with respect to $v$ values. Averaging across SNPs within genes and comparing joint effects retrieves a statistically significant joint effect between the two genes, *GCK* and *PAX4*, when comparing to the permuted null distribution. We can be confident that these results are not a result of overly large individual effects from the *GCK* or *PAX4* gene as the pairwise interaction ratio statistics used are with respect to the maximum of the marginal effects.

We take a moment to note that the number of SNPs corresponding to each gene varies among genes, ranging from 1 to 124 SNPs. On average there are roughly 3000 bp between two consecutive SNPs, which means the largest of our genes corresponds to more than 370,000 bp. We recognize the possibility of linkage disequilibrium between SNPs located close to one another. We take advantage of this dependence and integrate neighboring information by treating the gene as the basic unit instead of each SNP (thus accounting for our gene-based approach). Thus when we discuss the effect of a certain gene pair, we mean the average of all pairwise interactions of SNP pairs formed from the two genes.

## Conclusions

We find a significant interaction effect of the *GCK* and *PAX4* genes on hypertension in the GAW19 data. While *GCK* and *PAX4* have established coexpression and pathway linkages via other genes, no known interaction seems to have been previously established between the two genes themselves without the mediation of other genes. In addition, *GCK* and *PAX4* are not known to specifically interact toward hypertension. As such we provide direct evidence of an interesting joint effect of these two genes in the context of hypertension.
